# Clinical, imaging features and outcomes of patients with anti-GFAP antibodies: a retrospective study

**DOI:** 10.3389/fimmu.2023.1106490

**Published:** 2023-05-02

**Authors:** Bingqing Zhu, Mengyang Sun, Ting Yang, Haizhen Yu, Limei Wang

**Affiliations:** Department of Neurology, The First Affiliated Hospital of Zhengzhou University, Zhengzhou, Henan, China

**Keywords:** glial fibrillary astrocytic protein antibodies, clinical characteristics, imaging features, overlapping antibodies, prognosis

## Abstract

**Objective:**

To evaluate and compare the clinical features, imaging, overlapping antibodies, and prognosis of pediatric and adult patients with anti-GFAP antibodies.

**Methods:**

This study included 59 patients with anti-GFAP antibodies (28 females and 31 males) who were admitted between December 2019 and September 2022.

**Results:**

Out of 59 patients, 18 were children (under 18 years old), and 31 were adults. The overall cohort’s median age at onset was 32 years old, 7 for children, and 42 for adults. There were 23 (41.1%) patients with prodromic infection, 1 (1.7%) patient with a tumor, 29 (53.7%) patients with other non-neurological autoimmune diseases, and 17 (22.8%) patients with hyponatremia. Fourteen (23.7%) patients had multiple neural autoantibodies, with the AQP4 antibody being the most common. Encephalitis (30.5%) was the most common phenotypic syndrome. Common clinical symptoms included fever (59.3%), headache (47.5%), nausea and vomiting (35.6%), limb weakness (35.6%), and disturbance of consciousness (33.9%). Brain MRI lesions were primarily located in the cortex/subcortex (37.3%), brainstem (27.1%), thalamus (23.7%), and basal ganglia (22.0%). Spinal cord MRI lesions often involved the cervical and thoracic spinal cord. There was no statistically significant difference in the MRI lesion site between children and adults. Out of 58 patients, 47 (81.0%) had a monophasic course, and 4 died. The last follow-up showed that 41/58 (80.7%) patients had an improved functional outcome (mRS <3), and children were more likely than adults to have no residual disability symptoms (p = 0.001).

**Conclusion:**

There was no statistically significant difference in clinical symptoms and imaging findings between children and adult patients with anti-GFAP antibodies; Patients with anti-GFAP antibodies may present with normal MRI findings or delayed MRI abnormalities, and patients with overlapping antibodies were common. Most patients had monophasic courses, and those with overlapping antibodies were more likely to relapse. Children were more likely than adults to have no disability. Finally, we hypothesize that the presence of anti-GFAP antibodies is a non-specific witness of inflammation.

## 1 Introduction

Glial fibrillary acidic protein (GFAP) is an intermediate filament found primarily in astrocytes that serves as the skeleton of the cell and aids in cell communication and the formation of the blood-brain barrier. Abnormal regulation and expression of GFAP also play a key role in the onset and progression of various neurological diseases, including inflammation, traumatic brain injury, neurodegeneration, and so on (1–3). The Mayo Clinic (4) was the first to report a novel meningoencephalomyelitis with GFAP-IgG as a specific antibody that primarily affects the meninges, brain, spinal cord, and optic nerves in 2016. The condition was called autoimmune GFAP astrocytopathy (GFAP-A) (4). This neuroimmune disease has a distinct imaging feature known as paraventricular linear radial enhancement (4–7). The onset of this disease may be associated with a tumor or a viral infection, and it is frequently associated with overlapping antibodies (4, 5, 8–10). However, the French cohort questioned the existence of overlapping antibodies (11). Because the target antigen is intracellular, the pathogenicity of GFAP antibodies is debatable. The pathophysiological role of anti-GFAP antibodies in neuroimmunity is currently unknown. Despite various studies investigating the clinical characteristics and possible pathological features of patients with anti-GFAP antibodies, there is still no international consensus and guideline for diagnosis and treatment due to the disease’s heterogeneity. More diagnostic clues are required to develop early consensus on GFAP autoimmune diseases. This study aimsto describe the clinical characteristics, imaging, overlapping antibodies, and prognosis of pediatric and adult patients with anti-GFAP antibodies, as well as to speculate on the potential pathogenic mechanism of GFAP antibodies.

## 2 Materials and methods

### 2.1 Patients

From December 2019 to September 2022, we reviewed the medical records of 59 patients who had anti-GFAP antibodies in their serum or cerebrospinal fluid (CSF) and were consecutively admitted to the First Affiliated Hospital of Zhengzhou University. Inclusion criteria included (1): CSF or serum GFAP antibody-positive patients with one or more clinical manifestations of meningitis, encephalitis, myelitis, or optic neuritis (2); available clinical data; and (3) reasonable exclusion of other disorders Exclusion criteria include (1): patients with positive serum GFAP antibodies after traumatic brain injury or spinal cord injury (2); patients with glioma. Demographics, clinical manifestations, imaging, laboratory results, immunotherapy, disease course, and prognosis were all described. The modified Rankin Scale (mRS) was used to assess disease severity, and residual disability was followed up by phone. mRS < 3 was considered to be a good functional outcome.

### 2.2 Laboratory and imaging examination

Lumbar puncture was performed at least once on all patients. CSF white cell count, protein content, and oligoclonal bands (OCBs) were recorded at the earliest available time. Cell-based assays (CBA) were used to detect anti-GFAP antibodies in patient serum or CSF. Demyelinating antibodies (AQP4, MOG), autoimmune encephalitis-associated antibodies (such as NMDAR, GAD65, GABABR, LGI1, Caspr2, IGLON5, mGluR1, mGluR5, Hu, Ri, Yo, etc.) and systemic autoimmunity antibodies (such as RA, ANA, ANCA, dsDNA, CCP, SSA, SSB, etc.) were also detected. CSF from all patients was tested for viral, bacterial, and tuberculous bacteria to rule out CNS (central nervous system) infections. All patients had magnetic resonance imaging (MRI) of the brain or spinal cord performed at the time of admission on the same 3T MAGNETOM Skyra scanner (Siemens Healthcare, Erlangen, Germany), and some of them also received intravenous gadolinium to assess potential contrast enhancement. SE T1WI (TR = 488 ms, TE = 15 ms) and TSE T2WI (TR = 4000 ms, TE = 103 ms) sequences were used in transverse view, and T2WI - FLAIR (TR = 9000 ms, TE =81 ms) sequences in coronal view. The scanning matrix was 384 × 384, the field of vision was 230 mm×230 mm, the layer thickness was 6 mm, the slice gap was 1.2 mm, and the number of scanning layers was 18 ~ 20 layers. Spinal cord MRI scans were recorded using sagittal and transverse TSE T1WI and fat suppression sequence T1WI (cervical TR = 480 ms, TE = 9.4 ms; thoracolumbar TR = 337 ms, TE = 9.4 ms), TSE T2WI and fat suppression T2WI (cervical TR = 2700 ms, TE = 82 ms; thoracolumbar TR = 3500 ms, TE =87 ms), in which the cervical field of vision was 240 mm × 240 mm, thoracolumbar visual field was 340 mm × 340 mm, scan matrix was 384 × 384, the layer thickness was 3 mm, the slice gap was 0.3 mm, and the number of scanning layers was 15–18. The MRIs of the brain and spinal cord were reviewed by one neurologist and one neuroradiologist. A routine thyroid color ultrasound evaluation, abdominal color ultrasound, and chest CT examination were performed on all patients to rule out some common systemic tumors.

### 2.3 Standard protocol approvals

This study was ethically approved by the Ethics Committee of the First Affiliated Hospital of Zhengzhou University (2022-KY-1205–002).

### 2.4 Statistics

Patients were divided into two groups based on their age of onset: pediatric (<18 years old) and adult (≥18 years old). Statistical analyses and data visualization were performed using SPSS 26.0 and OriginPro 2021 to compare the clinical features and prognosis of pediatric and adult patients with anti-GFAP antibodies. To describe normally distributed continuous variables, means (standard deviation) were used. In contrast, for non-normally distributed continuous variables, the median (interquartile range) was used, and for categorical variables, the frequency (percentage) was used. The Wilcoxon rank-sum test (continuous variables) and the chi-squared test or Fisher exact test were used to compare two groups (categorical variables). P-values of <0.05 (two-sided) were considered to be statistically significant. Due to the exploratory nature of this study, we did not correct for multiple comparisons.

## 3 Results

### 3.1 General Conditions

This study included 59 patients (28 females and 31 males) who had anti-GFAP antibodies in their CSF or serum. Four patients (6.8%) were only positive for serum antibody, while the remaining patients had anti-GFAP antibody positive CSF with or without positive serum antibody. Furthermore, serum antibody titers were higher in four patients than CSF antibody titers (6.8%). The median duration of follow-up was 9 months (**Table 1**). The overall cohort’s median age at onset was 32, children aged 7 and adults aged 42. At the time of the first attack, 18 of the 59 GFAP-IgG-positive patients were under the age of 18. At the time of onset, only three patients (5.1%) were over 60 years old. The patient population in the other three age groups is comparable (20 patients in 0–20 years old, 15 patients in 21–40 years old, and 21 patients in 41–60 years old, respectively).

**Table 1:** Demographics and clinical features of GFAP-IgG patients.

Characteristic	Total	Pediatric patients	Adult patients	P
Number of patients, n	59	18	41	
Female:Male (% female)	28:31 (47.5%)	8:10 (44.4%)	20:21 (48.8%)	0.759
Age at onset, years, median (IQR)	32 (34.5)	7 (7.5)	42 (19.5)	
Follow-up, months, median (IQR)	9 (15)	12 (16)	9 (14)	
Comorbidity, n/total (%)				
Coexisting autoimmune diseases	29/54 (53.7%)	8/15 (53.3%)	21/39 (53.8%)	0.973
Tumor	2/59 (3.4%)	0	2/41 (4.9%)	1
Hyponatremia	17/59 (22.8%)	4/18 (22.2%)	13/41 (31.7%)	0.459
Monophasic course, n/total (%)	47/58 (81.0%)	16/18 (88.9%)	31/40 (77.5%)	0.508
Symptoms at presentation, n/total (%)				
Fever	35/59 (59.3%)	13/18 (72.2%)	22/41 (53.7%)	0.181
Headaches	28/59 (47.5%)	9/18 (50%)	19/41 (46.3%)	0.796
Nausea and vomiting	21/59 (35.6%)	7/18 (38.9%)	14/41 (34.1%)	0.726
Disturbance of consciousness	20/59 (33.9%)	6/18 (33.3%)	14/41 (34.1%)	0.952
Dizzy	14/59 (23.7%)	4/18 (22.2%)	10/41 (24.4%)	1
Psychiatric symptoms	8/59 (13.6%)	2/18 (11.1%)	6/41 (14.6%)	1
Cognitive deficits	7/59 (11.9%)	1/18 (5.6%)	6/41 (14.6%)	0.578
Seizure	5/59 (8.5%)	1/18 (5.6%)	4/41 (9.8%)	0.979
Impaired vision	6/59 (10.2%)	3/18 (16.7%)	3/41 (7.3%)	0.531
Diplopia	5/59 (8.5%)	2/18 (11.1%)	3/41 (7.3%)	1
Ataxia	3/59 (5.1%)	0	3/41 (7.3%)	0.546
Involuntary movement	7/59 (11.9%)	2/18 (11.1%)	5/41 (12.2%)	1
Optic disc edema	4/59 (6.8%)	1/18 (5.6%)	3/41 (7.3%)	1
Cranial nerve palsy	5/59 (8.5%)	2/18 (11.1%)	3/41 (7.3%)	1
Speech disorder	4/59 (6.8%)	0	4/41 (9.8%)	0.418
Walking unstable	4/59 (6.8%)	0	4/41 (9.8%)	0.418
Area postrema syndrome	2/59 (3.4%)	1/18 (5.6%)	1/41 (2.4%)	0.521
Weakness	21/59 (35.6%)	7/18 (38.9%)	14/41 (34.1%)	0.726
Numbness	10/59 (16.9%)	0	10/41 (24.4%)	0.055
Autonomic dysfunction	11/59 (18.6%)	4/18 (22.2%)	7/41 (17.1%)	0.418
Paresthesias	4/59 (6.8%)	0	4/41 (9.8%)	0.917
ICU admission	19/59 (32.2%)	7/18 (38.9%)	12/41 (29.3%)	0.466
Tracheal intubation	13/59 (22.0%)	2/18 (11.1%)	11/41 (26.8%)	0.317
mRS at the peak of attack, median (IQR)	4 (3)	3 (3)	4 (3)	0.692
mRS at discharge, median (IQR)	2 (2)	1 (2)	2 (2.5)	0.035
mRS score at the last follow-up, median (IQR)	1 (3)	0 (1.25)	1.5 (3)	0.003
mRS>2 at the last follow-up	17/58 (29.3%)	3/18 (16.7%)	14/40 (35%)	0.156
Sequelae, n/total (%)				
No disability	23/58 (39.7%)	13/18 (72.2%)	10/40 (25%)	0.001
Motor	17/58 (29.3%)	4/18 (22.2%)	13/40 (32.5%)	0.426
Sensory	12/58 (20.7%)	2/18 (11.1%)	10/40 (25%)	0.391
Vision impairment	6/58 (10.3%)	2/18 (11.1%)	4/40 (10%)	1
Autonomic dysfunction	6/58 (10.3%)	2/18 (11.1%)	4/40 (10%)	1
Cognitive impairment	5/58 (8.6%)	0/18	5/40 (12.5%)	0.288
Involuntary movement	2/58 (3.4%)	1/18 (5.6%)	1/40 (2.5%)	0.528
Speech disorder	2/58 (3.4%)	0	2/40 (5%)	1
Dysphagia	1/58 (1.7%)	0	1/40 (2.5%)	1
Death	4/58 (6.9%)	0	4/40 (10%)	0.406

IQR, interquartile range; ICU, intensive care unit; mRS, modified Rankin Scale.

23/56 (41.1%) patients had prodromic infection or vaccination before or at the time of onset, including 11/17 (64.7%) children and 12/39 (30.8%) adults. One patient had been immunized against COVID-19 one week before the onset of the disease. One patient had been infected with the varicella-zoster virus one month before the onset of neurological symptoms, and another had been infected with the herpes simplex virus (HSV) 2 weeks before. One patient had viral encephalitis one month prior, and the other had staphylococcal meningitis 2 weeks before the GFAP-IgG was discovered. A next-generation sequencing (NGS) test detected CSF infection in ten patients, including nine Human herpesviruses (Epstein-Barr virus n = 6, HSV n = 1, Human herpesvirus 7 n = 2) cases and one Staphylococcus case. There were also 4 cases with hepatitis B virus cases, 1 with tuberculosis, 1 with influenza B virus case, and 2 with mycoplasma cases. In all patients, only one (1.7%) was found to have a tumor, who was hospitalized with neurological symptoms and later diagnosed with papillary thyroid cancer. In addition, hyponatremia was present in 17/59 (22.8%) patients. Furthermore, 29/54 (53.7%) patients had other non-nervous system autoimmune diseases, with antibodies for these diseases, including anti-thyroid, antinuclear, antineutrophil cytoplasmic, antiphospholipid, rheumatoid factors, anti-dsDNA antibody, and Sjogren’s syndrome antibodies, among others. There was no statistically significant difference between children and adults in tumor, hyponatremia, or autoimmune antibodies (**Table 1**).

### 3.2 Clinical phenotype and clinical symptoms

**Figure 1:**
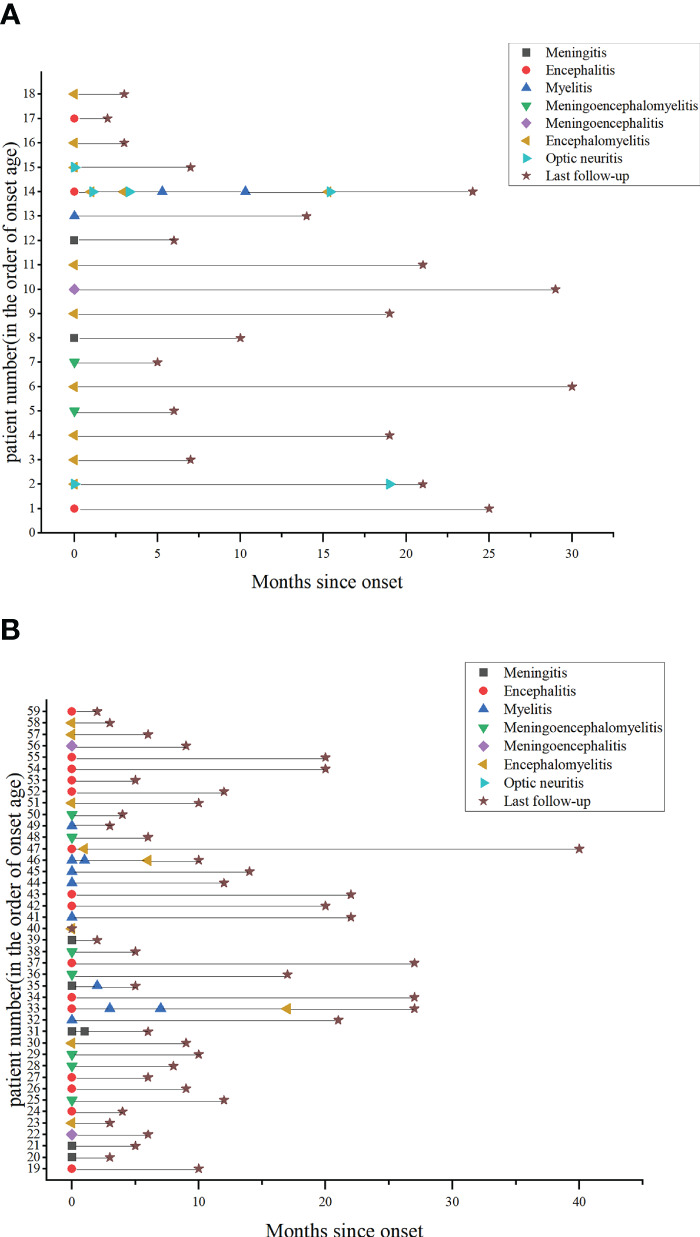
Clinical phenotype and disease course in patients with anti-GFAP antibodies. **(A)** Pediatric patients’ clinical phenotype and disease course. **(B)** Adult patients’ clinical phenotype and disease course.

The clinical course of 59 GFAP-IgG-positive children and adults is depicted in **Figure 1** to visually describe the clinical phenotypes of an acute attack. Among the 59 patients in the cohort, encephalitis (18/59, 30.5%) was the most common clinical phenotypic syndrome, followed by encephalomyelitis (15/59, 25.4%), meningoencephalomyelitis (10/59, 16.9%), meningitis (7/59, 11.9%), myelitis (7/59, 11.9%), meningoencephalitis (3/59, 5.1%) and optic neuritis (2/59, 3.4%). Encephalomyelitis (9/18, 50%) and encephalitis (15/41, 36.6%) were the most common clinical phenotypic syndromes in children and adults, respectively. Fever (59.3%), headache (47.5%), nausea and vomiting (35.6%), limb weakness (35.6%), disturbance of consciousness (33.9%), dizziness (23.7%), autonomic dysfunction (18.6%), and limb numbness were the most common clinical manifestations in the entire cohort (16.9%). Moreover, other clinical manifestations were cognitive impairment, involuntary movement, visual impairment, seizures, cranial nerve palsy, diplopia, optic disc edema, speech disorders, walking instability, ataxia, area postrema syndrome (APS), and paresthesia, etc. (**Table 1**). Clinical manifestations did not differ significantly between children and adults.

### 3.3 Cerebrospinal fluid analysis

CSF test results were available in 58 patients at the time of their initial presentation. Pleocytosis (> 5 cells/mm^3^) was found in 46 patients (79.3%), elevated protein level (> 0.5 g/L) in 35 patients (60.3%), and hypoglycorrhachia in 12 patients (20.7%). In the meantime, CSF-restricted OCBs (type 2) were found in 22 (40.7%) patients. CSF-elevated protein levels differed between children and adults (P = 0.005). (**Table 2**).

**Table 2:** Diagnostic testing and treatment of GFAP-IgG patients.

Characteristic	Value	Pediatric patients	Adult patients	P
CSF analysis at onset, n/total (%)				
Pleocytosis (> 5 cells/mm3)	46/58(79.3%)	15/18(83.3%)	31/40(77.5%)	0.875
Elevated protein level (> 0.5 g/L)	35/58 (60.3%)	6/18(33.3%)	29/40(72.5%)	0.005
Hypoglycorrhachia (<2.5 mmol/L)	12/58 (20.7%)	2/18(11.1%)	10/40(25%)	0.391
CSF Oligoclonal Bands (type 2)	22/54(40.7%)	6/14(42.9%)	16/40(40.0%)	0.851
Overlapping antibody n/total (%)				
AQP4-IgG	7/59(11.9%)	2/18(11.1%)	5/41(12.2%)	
MOG-IgG	5/59(8.5%)	3/18(16.7%)	2/41(4.9%)	
NMDAR-IgG	3/59(5.1%)	1/18(5.6%)	2/41(4.9%)	
Others*	3/59(5.1%)	0/18	3/41(7.3%)	
MRI, n/total (%)				
MRI (brain) abnormalities	43/59(72.9%)	16/18(88.9%)	27/41(65.9%)	0.130
Gadolinium enhanced lesion (brain)	23/40(57.5%)	5/7(71.4%)	18/33(54.5%)	0.689
Leptomeninges enhancement	11/40(27.5%)	2/7(28.6%)	9/33(27.3%)	1
Perivascular-radial enhancement	4/40(10.0%)	0	4/33(12.1%)	1
Lesion location				
Juxtacortical	22/59(37.3%)	8/18(44.4%)	14/41(34.1%)	0.451
Periventricular	9/59(15.3%)	2/18(11.1%)	7/41(17.1%)	0.847
Corpus callosum	9/59(15.3%)	4/18(22.2%)	5/41 (12.2%)	0.553
Basal ganglia	13/59(22.0%)	4/18(22.2%)	9/41 (22.0%)	1
Thalamus	14/59(23.7%)	6/18(33.3%)	8/41 (19.5%)	0.414
Brachium pontis	4/59(6.8%)	1/18(5.6%)	3/41(7.3%)	1
Brainstem tegmentum	16/59(27.1%)	3/18(33.3%)	10/41(24.4%)	0.751
Cerebellar hemispheres	5/59(8.5%)	2/18 (11.1%)	3/41(7.3%)	1
MRI (spinal cord) abnormalities	35/50(70.0%)	13/16(81.3%)	22/34(64.7%)	0.390
Gadolinium enhanced lesion (spinal cord)	13/21(61.9%)	3/3(100%)	10/18(55.6%)	0.409
LETM	17/50(34.0%)	8/16(50.0%)	9/34(26.5%)	0.101
Cervical cord	26/50 (52.0%)	10/16(62.5%)	16/34(47.1%)	0.308
Thoracic cord	24/50(48.%)	10/16(62.5%)	14/34(41.2%)	0.159
Medullary cone	2/50(4.0%)	2/16(12.5%)	0/34	0.098
Acute phase treatment n/total (%)				
IVMP alone	26/59(44.1%)	5/18(27.8%)	21/41(51.2%)	
IVMP+IVIG	22/59(37.3%)	11/18(61.1%)	11/41(26.8%)	
Others^	7/59(11.9%)	2/18(11.1%)	5/41(12.2%)	
No immunotherapy	4/59(6.8%)	0	4/41(9.8%)	
Maintenance therapy n/total (%)				
Glucocorticoids alone	40/59(67.8%)	15/18(83.3%)	25/41(61.0%)	
Mycophenolate mofetil with or without glucocorticoids	9/59(15.3%)	1/18(5.6%)	8/41(19.5%)	
Others #	2/59(3.4%)	1/18(5.6%)	1/41(2.4%)	

*: GAD65-IgG, Yo-IgG, GlyR-IgG

^:IVMP+RTX,n = 2;IVMP+PE+IVIG,n = 2;IVMP+IVIG+RTX,n = 1;IVMP+PE,n = 1;IVIG+EIA,n = 1.

#: tacrolimus in one adult patients, azathioprine in one pediatric patient

CSF, cerebrospinal fluid; AQP4, Aquaporin 4; MOG, Myelin oligodendrocyte glycoprotein; NMDAR, N-methyl-D-aspartate receptor; MRI, magnetic resonance imaging; LETM, longitudinally extensive transverse myelitis; IVIG, intravenous immunoglobulin; IVMP, intravenous methylprednisolone; RTX, rituximab; PE, plasma exchange; EIA, extracorporeal immunoadsorption.

### 3.4 Imaging manifestations

**Figure 2:**
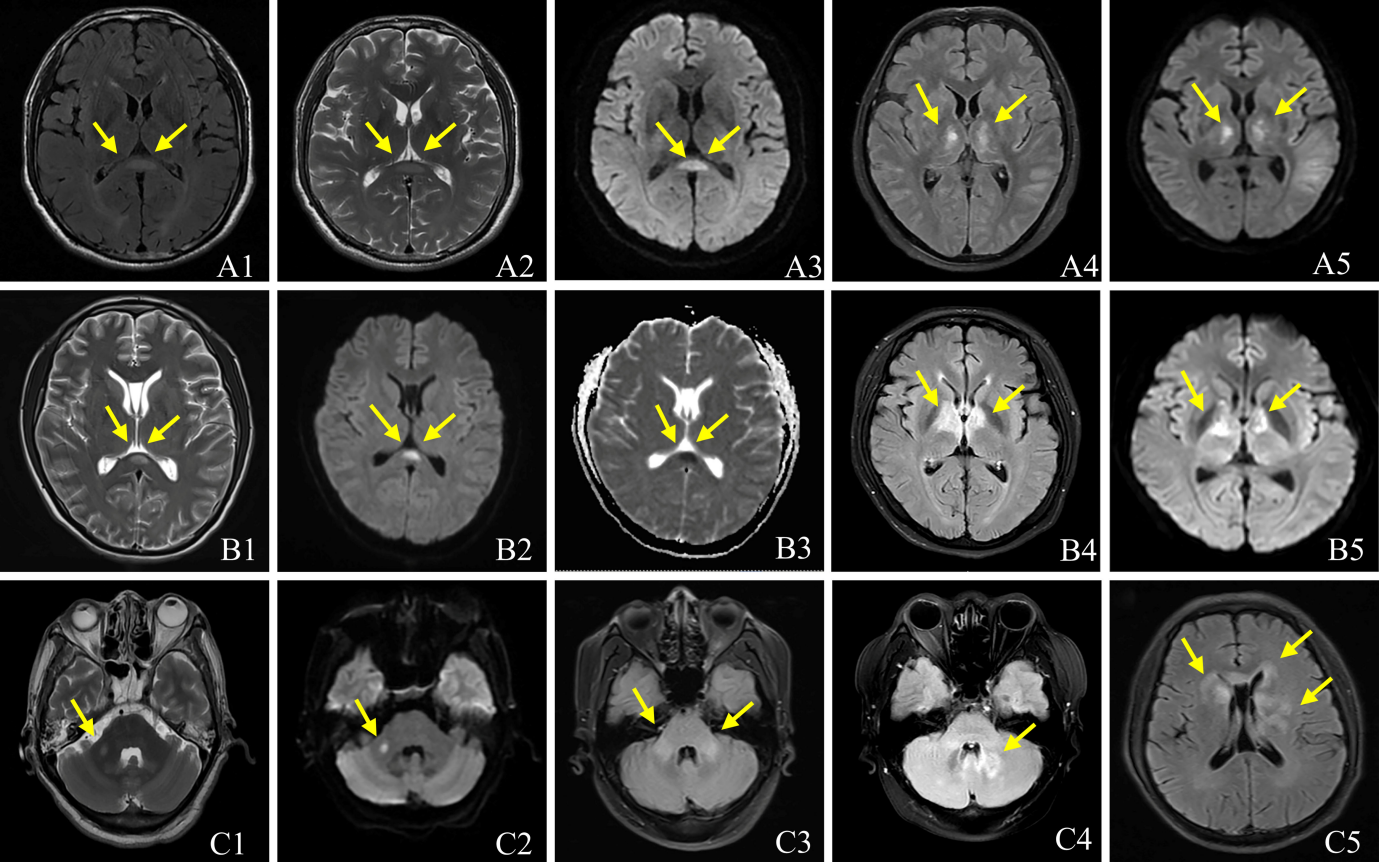
Brain MRI characteristics of patients with Anti-GFAP antibodies. The imaging looks like a reversible splenial lesion **(A1-A3, B1-B3)**. T2 FLAIR **(A4, B4)** and Diffusion Weighted Imaging **(A5, B5)** show lesions in bilateral thalamus, T2-weighted **(C1)** image and FLAIR **(C2, C3)** show lesions in brachium pontis; T2 FLAIR shows lesions in cerebellum **(C4)**, basal ganglia **(C5)** and paraventricular **(C5)**.

**Figure 3:**
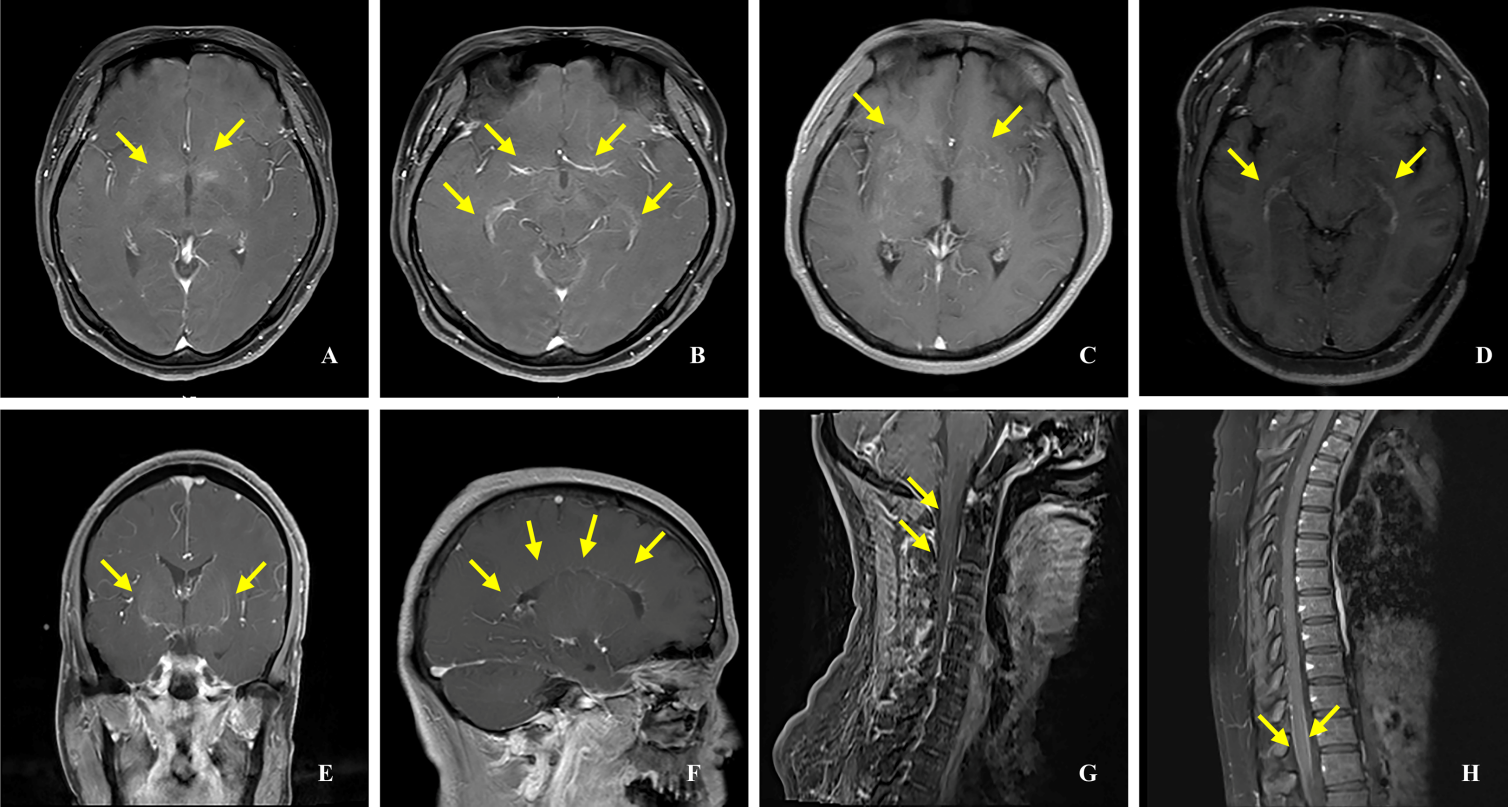
Gadolinium enhancement MRI in patients with anti-GFAP antibodies. **(A, C)** Punctate enhancement lesions. **(B, D)** Periependymal enhancement. **(E)** Linear enhancement of the cerebellum. **(F)** Linear enhancement perpendicular to the ventricle. **(G)** Linear enhancement of the spinal cord. **(H)**Enhancement of the spinal cord membranes.

**Figure 4:**
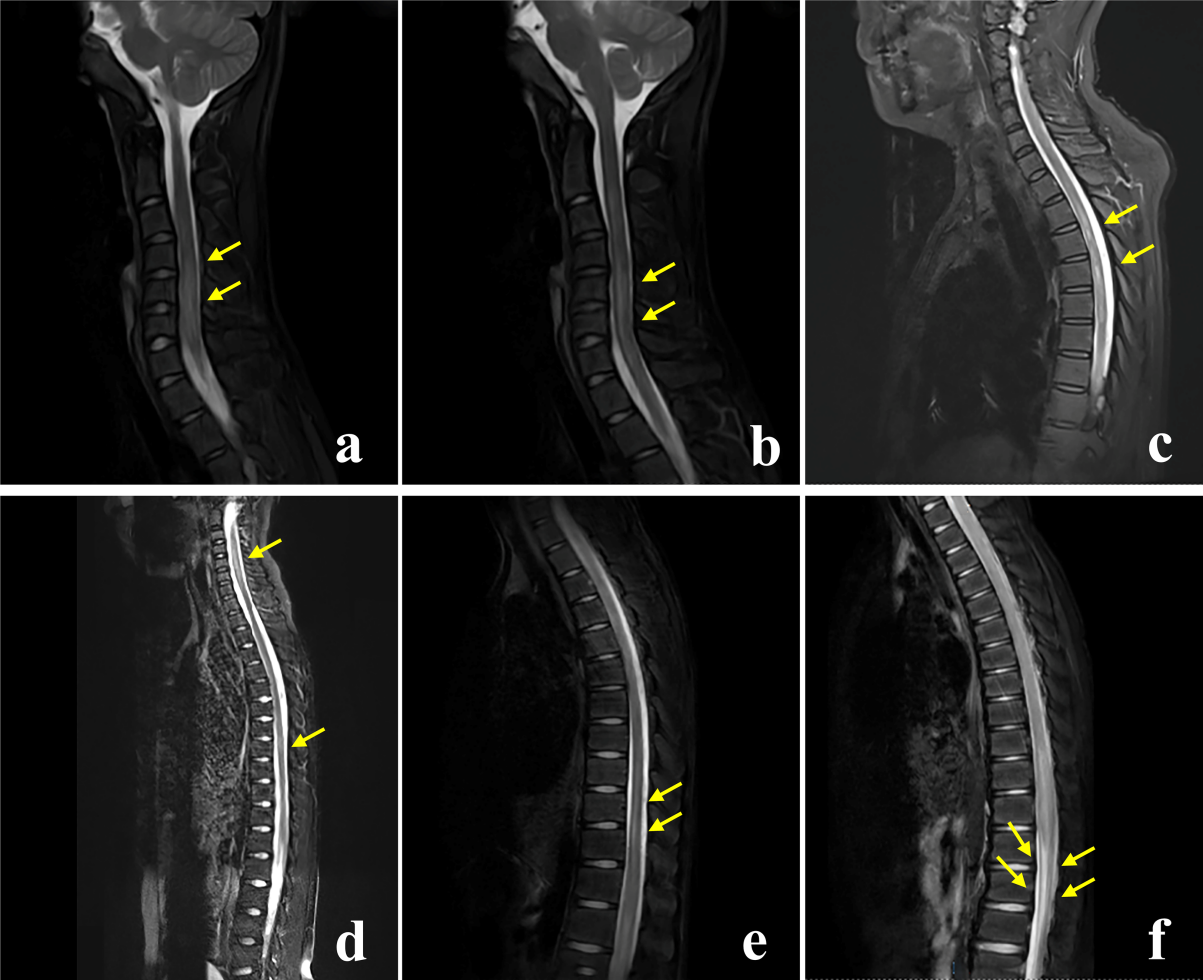
Spinal cord MRI characteristics of patients with Anti-GFAP antibodies. The MRI T2-weighted fat suppression sequence of the spinal cord in patients with anti-GFAP antibodies shows that the morphology of spinal cord lesions could be long-segment patchy lesions **(A, B)**, multiple short-segment lesions **(C)**, and long-segment linear lesions **(D, E)**. Abnormal signal of conus medullaris in 1 patient **(F)**.

All patients underwent brain MRIs. During the acute phase, 43 patients (72.9%) had abnormal brain MRIs, displaying hyperintensities on T2-weighted and fluid-attenuated inversion recovery (FLAIR) sequences (**Figure 2**). Five patients’ brain MRIs were normal at the start of their symptoms but gradually became abnormal. In four patients with clinical manifestations, the brain MRIs revealed no lesions. The most common lesions were in the cortical/subcortical (37.3%), brainstem (27.1%), thalamus (23.7%), basal ganglia (22.0%), periventricular (15.3%), and corpus callosum (15.3%). The cerebellar hemisphere (8.5%) and the pontine arm (6.8%) were also unusual sites (**Table 2**). One of the most common imaging features was lesions in the bilateral thalamus (20.3%) and bilateral basal ganglia (18.6%) (**Figure 2**). In 6/59 (10.2%) patients, reversible splenial lesion syndrome (RESLES) was discovered. Among the 40 patients who underwent brain gadolinium enhancement MRI, 23 had enhanced lesions, 11 had leptomeningeal enhancement, but only 4 patients had periventricular or cerebellar linear enhancement (**Figure 3**). There were 50 patients with spinal cord MRI, 35 (70.0%) of whom had abnormal signals (**Figure 4**), and 17 (34.0%) had a longitudinal extension to more than three adjacent vertebral segments (longitudinal extensive transverse myelitis, LETM). Cervical, thoracic, and spinal conus lesions were responsible for 26/50 (52.0%), 24/50 (48.0%), and 2/50 (4.0%) of the cases, respectively. The lumbar spinal cord was free of lesions. In 21 patients, enhanced MRI of the spinal cord was performed, and 13 cases were found to have enhanced lesions, including two cases of spinal membrane enhancement and one case of cauda equina nerve enhancement. **Table 2** shows that there is no statistically significant difference in the MRI lesion site between children and adults.

### 3.5 Overlapping antibodies

Four males (28.6%) and ten females (71.4%) were among the 59 patients who coexisted with other neural autoantibodies (**Table 2**). **Table 3** shows the information on patients who have overlapping antibodies. Four of fourteen patients (28.6%) with overlapping antibodies relapsed. Six of the 14 patients (14, 15, 32, 41, 46, and 52) were tested positive for AQP4 antibody in serum, GFAP and AQP4 antibody in CSF. Four patients with positive MOG antibodies were combined separately (patients 2, 6, 24, and 33, GFAP and MOG in serum, GFAP in CSF).

**Table 3:** Clinical, imaging, treatment, and prognosis of patients with GFAP overlapping antibodies.

Patient no. Sex/age (y.)	Neural autoantibodies in serum	Neural autoantibodies in CSF	Symptoms	lesion location in MRI	Treatment	Disease course	mRS at last follow-up
14. F/12	AQP4	GFAP/AQP4	Fever, vomiting, stagger, central facial paralysis blurred vision, lower extremity weakness, involuntary movement, hearing loss, numbness and weakness of limbs	Pons, medulla oblongata, C1–5, C6–7	IVMP+IVIG+RTX	Relapse	1
15.F/14	AQP4	GFAP/AQP4	Fever, headache, blurred vision	Paraventricular third ventricle, periaqueductal gray, basal ganglia, C2–3, T4–5, T8	IVMP	Monophasic	0
32.F/35	AQP4	GFAP/AQP4	Numbness of limb, paroxysmal limb twitch	Medulla oblongata, pons, C1-C6,T2-T5	IVMP	Monophasic	1
41.F/44	AQP4	GFAP/AQP4	Paroxysmal limb twitch, paresthesias	C1-T1	IVMP+IVIG	Monophasic	1
46.F/48	AQP4	GFAP/AQP4	Fever, weakness and numbness in both lower extremities, dysuria	C2-T8 (multiple focal lesion)	IVMP+RTX	Relapse	4
52.F/55	AQP4	GFAP/AQP4	Nausea and vomiting, dysphagia, facial pain, numbness of limb	Dorsal medulla oblongata, hippocampus, basal ganglia, periventricular	IVMP	Monophasic	3
2.F/3	GFAP/MOG	GFAP/MOG	Fever, blurred vision	Bilateral frontal, parietal and temporal lobes, splenium of corpus callosum, pons, right cerebellar hemisphere, C4-T8	IVMP	Relapse	2
6.M/5	GFAP/MOG		Headache, double vision, dizziness, lethargy,	Bilateral thalamus and ganglia, bilateral parietal, temporal and occipital lobes, brainstem C5-T12	IVMP+IVIG	Monophasic	0
24.F/26	GFAP/MOG	GFAP	Fever, headache, nausea and vomiting, double vision, lower limb weakness, seizure	Bilateral cerebellar hemispheres, bilateral thalamus	IVMP	Monophasic	1
33.M/36	GFAP/MOG	GFAP	Headache, blurred vision numbness, weakness	Frontal cortex, subcortex, left thalamus, cerebral peduncle, around the fourth ventricle, bilateral cerebellar hemispheres, C1-T3	IVMP	Relapse	1
13.F/11	GFAP/NMDAR/MOG	GFAP/NMDAR/MOG	Lower extremity weakness, hypersomnia	C2–6, T9–12	IVMP	Monophasic	0
27.F/31	GFAP/AQP4	NMDAR/GAD65/GlyR	Headache, dizziness, hypersomnia, disturbance of consciousness	Optic chiasma, bilateral thalamus, fornix column, and third ventricle area	IVMP+IVIG	Monophasic	1
30.M/32	Yo	GFAP	Dizziness, stagger, limb weakness	Normal	NA	Monophasic	2
36.M/41		GFAP/NMDAR	Fever, hypersomnia, delirium, lower extremity weakness, disturbance of consciousness	Leptomeninge enhancement in bilateral cerebral hemispheres and brain stem surface,T1–7	NA	Monophasic	0

F, female; M, male; C, cervical spinal cord; T, thoracic spinal cord; IVMP, intravenous methylprednisolone; IVIG, intravenous immune globulin; RTX, rituximab; mRS, modified Rankin Scale; NA, not available; MRI, magnetic resonance imaging.

The first symptoms of Patient 14 were nausea and vomiting, which were quickly followed by fever, shaky walking, central facial paralysis, blurred vision, and limb weakness. Despite various immunotherapies, the condition recurred six times. At the last follow-up, Patient 46 still had weakness in both lower limbs due to recurrent myelitis-like symptoms. With dysarthria, asphyxia, blurred vision, and other symptoms, Patient 52 was discharged. Patient 2 presented with a fever and blurred vision in the right eye and was given methylprednisolone intravenously (IVMP). The symptoms were completely resolved at discharge and were treated with oral glucocorticoids and mycophenolate mofetil. The patient, however, lost vision in his left eye three months after glucocorticoids withdrawal and was discharged with visual impairment. Patient 6 was admitted to the hospital for two days with the chief complaint of headache, diplopia, and low spirits. He might have had viral encephalitis a month before. His symptoms completely resolved after IVMP combined with IVIG treatment, and he was discharged and diagnosed with acute disseminated encephalomyelitis (ADEM). The clinical manifestations of patient 30 were dizziness, unsteady walking, and limb weakness (anti-Yo antibodies in serum, anti-GFAP antibodies in CSF). Cerebellar and brainstem inflammation was diagnosed based on the clinical symptoms, but brain MRIs revealed no obvious abnormalities. The patient was later transferred to a nearby hospital and was still having difficulty walking at the time of the last check-up. In one patient (patient 36), GFAP and NMDAR antibodies were found in the CSF, as well as fever, lower limb weakness, and confusion. With an mRS score of 5, the patient was admitted to the ICU, and his family refused IVMP combined with IVIG treatment, requesting transfer to another hospital for treatment. The patient had fully recovered at the time of the last check-up.

### 3.6 Treatment, outcome, and follow-up

During the course of the disease, 19 (32.2%) and 13 (22.0%) patients were admitted to ICU and intubated, respectively. Two patients’ families refused immunotherapy, and another two patients did not receive immunotherapy because they were diagnosed with clinically isolated syndrome (CIS) and cerebral infarction, respectively. Only 55 patients received acute-phase immunotherapy. 26/55 patients received IVMP alone, 22/55 received IVMP in combination with IVIG, and 7/55 received other combination immunotherapies.

**Figure 5:**
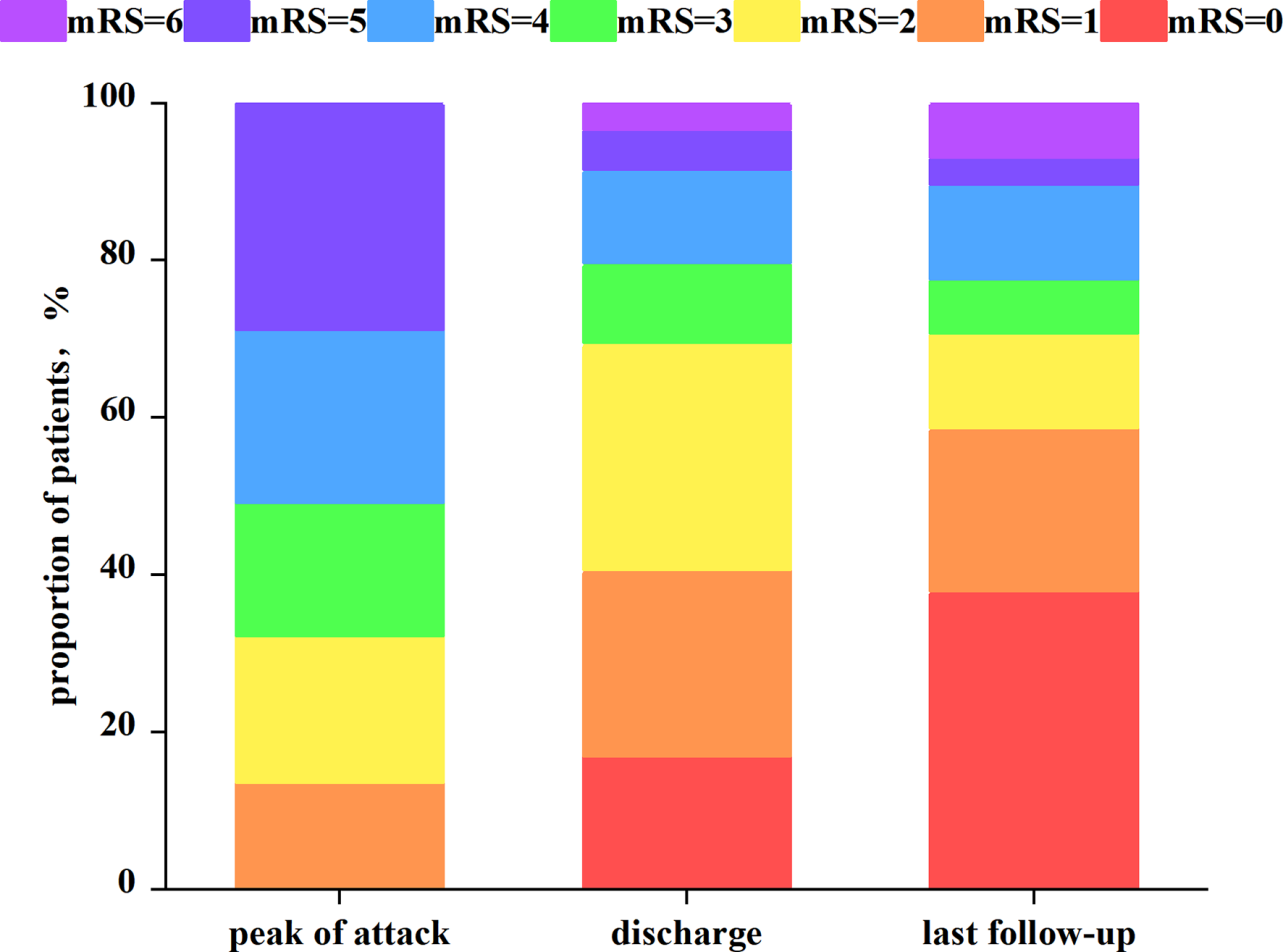
Distribution of the modified Rankin Scale score at the peak of the attack, discharge, and the last follow-up.

Patients were contacted by phone in all cases, except one, who was missed due to a change in the phone number. During the follow-up period, 49 patients received oral glucocorticoids and were tapered, with 11 receiving additional immunosuppressive drugs (mycophenolate n = 9, azathioprine n = 1, tacrolimus n = 1). **Figure 5** depicts the mRS distribution of the 59 patients at the peak of the attack, discharge, and the last follow-up. There were significant differences in mRS scores between children and adults at discharge (p = 0.035) and at the last follow-up (p = 0.003) 41/58 (80.7%) patients had good functional outcomes at the last follow-up (mRS<3). Recurrence occurred in 7/58 (12.1%) patients (2 children and 5 adults), with 4 patients recurring only once, 1 patient recurring twice (coexistence of AQP4-IgG), 1 patient recurring three times (coexistence of MOG-IgG), and 1 patient recurring six times (coexistence of AQP4-IgG). There were no residual symptoms in 23/58 patients (39.7%), including 13/18 (72.2%) children and 10/40 (25%) adults, which was statistically significant (p = 0.001). At a median of 9 months (range 0–40 months), 31/58 (53.4%) patients had residual symptoms. The most common type of disability was myelitis-like symptom. Blurred vision, cognitive dysfunction, involuntary movement, slurred speech, and dysphagia were among the other uncommon disabilities. Four patients (6.9%) died between the onset of the disease and the last follow-up.

## 4 Discussion

Our study included 59 patients with anti-GFAP antibodies in CSF or serum (patients with the meningoencephalomyelitis phenotype and excluding other diagnoses) to compare clinical characteristics, imaging, overlap antibodies, and prognosis in pediatric and adult patients, which has been rarely reported in previous studies.

In this study, the proportion of male and female patients was roughly equal older patients were less likely to be affected. Patients in our cohort frequently presented with symptoms of meningitis, encephalitis, myelitis, and optic neuritis. Non-specific symptoms such as fever, headache, nausea, and vomiting, as well as myelitis-like symptoms and consciousness disturbance, are common clinical manifestations. Monophasic course (81.0%) was common, whereas patients with overlapping antibodies were more likely to relapse, especially when combined with AQP4 and MOG antibodies. Four patients had symptoms of speech dysfunction, which had been rarely reported in earlier studies. Two patients developed APS, as previously reported (12), implying that APS should not only be considered as a diagnosis of neuromyelitis optica spectrum disorders (NMOSD) but should also be tested for anti-GFAP antibodies.

Some patients only with anti-GFAP antibodies in serum were included in this study because they presented with symptoms of autoimmune GFAP-A and ruled out other diagnoses. Antibody titers in serum were higher in some patients than in CSF, contradicting previous reports. A higher serum titer than CSF indicates that antibodies may have originated in the peripheral blood system. In contrast, a higher CSF than serum indicates that antibodies may have originated in the CNS via intrathecal synthesis. In our study, 40.7% of patients have CSF-restricted OCBs, which is an indicator of intrathecal synthesis. Therefore, it is reasonable to think that anti-GFAP antibodies may originate in different places. In light of these findings, we should look further into the pathogenic mechanisms and the source of anti-GFAP antibodies.

Some patients in this study had a history of herpes virus infection before the onset of neurological symptoms, whereas others had Epstein-Barr virus and herpes virus infection detected by CSF samples using NGS technology at the onset. Precursor infections are more common in children, possibly because the blood-brain barrier is not fully developed in some children. A previous study reported the first case of autoimmune GFAP-A following HSV encephalitis infection and proposed that HSV infection might activate the immune response to autoimmune GFAP-A (13). Infection appears to be associated with the pathogenesis of GFAP astrocytopathy but the neuroimmune mechanism that infection activates is unknown. One possible mechanism is that the infection damaged the astrocytes, exposing and releasing many GFAP antigenic determinants, leading to antibody production and secondary autoimmune responses. Another possibility is that some infectious pathogen components, such as amino acids, have a sufficiently similar structure or sequence to the host’s GFAP antigen. The immune response to pathogen antigen may have an impact on the host’s GFAP antigen. Therefore, we recommend testing for GFAP antibodies in patients with viral encephalitis who do not respond to antiviral therapy and checking for CSF infection status in patients with anti-GFAP antibodies. Furthermore, previous research in other countries has found that 12-38% has tumors (4, 5, 11, 14), and the occurrence of tumors may be associated with the production of GFAP antibodies. However, when compared to other studies, the incidence of tumors in our study is low. According to a Mayo Clinic study (5), 66% of tumors are detected within two years of the onset of symptoms, so the variability associated with tumors could be due to the study’s small sample size and short follow-up time. On the other hand, a previous Chinese report (6) found no concomitant tumor, which is consistent with our findings. Therefore, we believe that tumor-related differences are more likely to be racial.

This study discovered that patients with anti-GFAP antibodies frequently had pathological findings in their CSF, including elevated cell counts and proteins. Hyponatremia occurs in some patients during the course of the disease, possibly due to thalamic lesion involvement. As a result, hypothalamic function is impaired, and normal mechanisms that regulate the secretion of antidiuretic hormones are disrupted. Furthermore, like those with NMOSD, these patients frequently have other systemic autoimmune diseases.

In previous studies, approximately half of GFAP antibody-positive patients had specific imaging findings of paraventricular linear radial enhancement. In contrast, in our study, only 4/40 (10%) patients had linear perivascular enhancement oriented to the ventricle, while leptomeningeal enhancement was more common (4, 5, 7, 15). Furthermore, lesions on brain MRI were mostly found in the cortex/subcortex, brainstem, thalamus, and basal ganglia. Lesions in the bilateral thalamus (20.3%) and basal ganglia (18.6%) were among the most frequent features and also matched with the findings from a Japanese study, including 14 participants (16). The cervical and thoracic spinal cords are frequently involved in spinal cord MRI lesions, but the lumbar spinal cord is rarely involved. Furthermore, LETM (34.0%) was more common in the cohort, which was consistent with previous research findings (5, 6). This study included five patients who initially presented with neurological symptoms without abnormalities on MRI but later developed radiographic lesions. Previous research have found that initial brain MRI reveals non-specific findings, but the brain MRI in reexamination and follow-up reveals characteristic autoimmune GFAP-A findings (17, 18). A case report also suggested that there was some light meningeal enhancement at first, followed by the gradual development of multiple intracranial lesions (19). These findings suggest that MRI abnormalities may delay the appearance of autoimmune GFAP-A and that MRI examinations may need to be repeated to properly diagnose this disease. Six patients with RESLES were identified on brain MRI in this study, which has previously been reported in autoimmune GFAP-A (11, 20, 21). RESLES is a rare clinical-radiographic disease with unknown pathogenesis. According to the French cohort, this unique MRI performance may support the hypothesis that GFAP autoimmunity is triggered by infection (11). In conjunction with this study, we consider RESLES to be a specific clinical imaging finding of GFAP-A, implying that patients with RESLES should also be considered for a diagnosis of GFAP autoimmune disease. Four patients in this study (three children and one adult) had clinical signs of neurological disease, but MRIs of the brain and spinal cord were normal. Previous studies have also reported on this occurrence (5, 14, 21). Previous research has suggested that normal MRI findings may be a common outcome in children, which is consistent with our findings (21). This phenomenon suggests that autoimmune GFAP-A should be considered in patients (particularly children) who have meningoencephalomyelitis-like clinical manifestations but no MRI abnormalities.

Several studies have shown that overlapping antibodies are common in autoimmune GFAP-A (5, 6, 8, 10, 22). 14/59 (23.7%) patients in our study had overlapping antibodies. NMDAR-IgG was the most common coexisting antibody in a Mayo Clinic study of 102 patients with autoimmune GFAP-A, followed by AQP4-IgG (5). Two Chinese studies discovered that AQP4-IgG and MOG-IgG were the most common coexisting antibodies (8, 22). The most common coexisting antibody in this study was AQP4-IgG, followed by MOG-IgG and NMDAR-IgG. Interestingly, our study is the first to show a specific multi-antibody overlaps: GFAP-IgG and AQP4-IgG in serum, NMDAR-IgG, GAD65-IgG, and GlyR-IgG in CSF. Although coexisting of MOG-IgG and AQP4-IgG were found in a French cohort study, simultaneous involvement of MOG-IgG in the peripheral nervous system was thought to be unusual, and AQP4-IgG was only found in CSF, casting doubt on the existence of an overlap syndrome in GFAP autoimmunity. The finding in the French cohort contradicts our findings, which is thought to be because some studies in China have found that AQP4-IgG is the most common coexisting antibody, and the detection rate of AQP4 antibodies in Asian populations is higher than in Caucasian populations. The incidence and prevalence of NMOSD vary greatly by ethnicity and region, with Asians being particularly vulnerable. To summarize, the precise mechanism underlying the occurrence of overlapping antibodies is unknown, and how to correctly diagnose and classify patients with autoantibody overlapping syndrome is a problem that must be solved in the future. Two Mayo Clinic studies (5, 14) discovered that the presence of both GFAP-IgG and NMDAR-IgG at the same time was associated with an increased risk of tumors. However, no tumor was observed in this study when GFAP-IgG coexisted with NMDAR-IgG, which may be due to ethnic specificity, as tumors were rare in Chinese patients with anti-GFAP antibodies. At the moment, there is no clear pathogenesis for the co-occurrence of antibodies, and determining which antibodies are pathogenic is difficult. GFAP is an intracellular protein antigen, unlike AQP4, NMDAR, MOG, and other cell surface antigens, and its antibody cannot be directly contacted to produce humoral immunity. Furthermore, previous animal studies (23) have demonstrated that CD8 T cells targeting GFAP in the CNS can avoid tolerance mechanisms and cause gray and white matter lesions in the brain and spinal cord. How CNS-reactive CD8T cells are activated determines the clinical and histological characteristics of lesions. That is, spontaneously recruited GFAP-specific CD8T cells to infiltrate the CNS gray and white matter, resulting in relapse remission and chronic CNS autoimmunity. In contrast, virus-induced GFAP-specific CD8 T effector cells specifically target the meninges and vascular/perivascular spaces of gray matter and white matter, resulting in rapid, acute CNS disease. This pathogenic mechanism fits the disease course and clinical characteristics of autoimmune GFAP-A. Anti-GFAP antibodies may not be pathogenic, but they do serve as a marker of autoimmunity caused by cytotoxic T cells (4, 24).

According to a 2018 study, the immunopathological manifestations of GFAP astrocytic lesions were astrocyte and neuron loss (6). Another study, however, discovered that a patient with positive CSF GFAP antibody had no astrocyte involvement or demyelination in the autopsy and speculated that GFAP antibody was not the pathogenic antibody causing astrocyte inflammation but rather a bystander autoantibody of inflammation (25). The majority of patients in our study had other neuronal surface antibodies or viral infections, implying that GFAP antibodies might be a non-specific witness of inflammation. At the moment, the pathogenicity of GFAP autoantibodies is debatable, and more pathological evaluations are required to determine whether they are pathogenic.

The majority of patients in this study responded well to immunotherapy and were improved by the time they were discharged. Furthermore, the majority of patients had a good functional outcome, with 37.9% completely asymptomatic at the last follow-up (mRS = 0). Notably, 29.3% of patients still had poor functional outcomes (mRS > 2), including four patients (all adults) who died, indicating that immunotherapy did not work for all patients (26). Children were more likely than adults to have no residual disability at the last follow-up, implying that age may influence patient outcomes. Based on the foregoing, we can conclude that some patients have poor prognostic outcomes, and future research should look into the factors influencing poor prognosis. Furthermore, our patients were frequently diagnosed with viral encephalitis, tubercular meningitis, ADEM, and even CIS during the course of the disease, as previously reported (27–30), indicating that we should improve the relevant diagnostic criteria of GFAP autoimmune diseases and develop standardized treatment methods as soon as possible.

## 5 Conclusion

In conclusion, older patients were less likely to be affected, and male and female patients were roughly equally represented. Patients with anti-GFAP antibodies are often complicated with infection, autoimmunity, hyponatremia, and pathological CSF. In the meantime, patients with overlapping antibodies are common; however, the mechanism of overlapping antibodies and pathogenic antibodies is unknown. Tumors were discovered in a small number of patients. Patients frequently present with one or more of the following symptoms: encephalitis, meningitis, myelitis, and optic neuritis. Lesions on brain MRI are frequently found in the cortex/paracortex, brainstem, thalamus, and basal ganglia. One of the hallmark imaging findings of this disease may be bilateral thalamic and basal ganglia lesions. MRI lesions of the spinal cord are most commonly found in the cervical and thoracic medulla. In a small number of patients, MRI abnormalities may delay the appearance of autoimmune GFAP-A, or the MRI finding may be normal, or they may present with RESLES at the onset of autoimmune GFAP-A. Clinical manifestations and imaging findings did not differ significantly between children and adults with anti-GFAP antibodies. The majority of patients had a monophasic course, and those with overlapping antibodies were more likely to relapse. The majority of patients respond well to immunotherapy and have a good prognosis, but a few have a poor prognosis, such as death. Some patients may be misdiagnosed as having viral encephalitis, tuberculous encephalitis, ADEM, CIS, and other conditions. Children are more likely than adults to have no disability. Finally, we hypothesized that the presence of GFAP antibodies was a non-specific witness of inflammation.

## 6 Limitations

There are some limitations to this study. Firstly, the presented data were retrospectively obtained from an electronic medical record system. Secondly, the sample size was small, and the data were collected from a single center. Finally, selection bias might exist because the First Affiliated Hospital of Zhengzhou University is a tertiary referral center.

Patients are frequently misdiagnosed as having tuberculous meningitis, ADEM, or viral encephalitis due to a lack of knowledge about the disease, resulting in late and incorrect treatment. Therefore, we should develop early guidelines for diagnosing and treating autoimmune GFAP-A in collaboration with colleagues both at home and abroad. In addition, multicenter and large-sample clinical studies with long-term follow-up are suggested to identify factors associated with relapse and poor prognosis in patients with anti-GPAP antibodies in the future.

## Data availability statement

The raw data supporting the conclusions of this article will be made available by the authors, without undue reservation.

## Ethics statement

The study was performed in adherence to ethical guidelines and was ethically approved by the Ethics Committee of The First Affiliated Hospital of Zhengzhou University (2022-KY-1205–002).

## Author contributions

BZ and LW conceived and designed the study. BZ drafted the manuscript. MS and HY collected and analyzed the data. TY reviewed and edited the manuscript. LW revised the manuscript critically.
